# Range Analysis and Terrain Preference of Adult Southern White Rhinoceros (Ceratotherium simum) in a South African Private Game Reserve: Insights into Carrying Capacity and Future Management

**DOI:** 10.1371/journal.pone.0161724

**Published:** 2016-09-13

**Authors:** S. Thompson, T. Avent, L. S. Doughty

**Affiliations:** Spatial Ecology and Landuse Unit, Oxford Brookes University, Headington, Gipsy Lane, Oxford, OX3 0BP, United Kingdom; University of Sydney, AUSTRALIA

## Abstract

The Southern white rhinoceros (*Ceratotherium simum*) is a threatened species, central to the tourism appeal of private game reserves in South Africa. Privately owned reserves in South Africa tend to be smaller than government run reserves such as Kruger National Park. Because of their relatively small size and the often heterogeneous nature of the landscape private game reserve managers benefit from detailed knowledge of white rhinoceros terrain selection preferences, which can be assessed from their ranging behaviours. We collected adult and sub-adult white rhinoceros distribution data over a 15 month period, calculating individual range size using kernel density estimation analysis within a GIS. From this, terrain selectivity was calculated using 50% and 95% kernels to extract terrain composition values. Jacob’s correction of the Ivlev’s selectivity index was subsequently applied to the terrain composition of each individual to identify trends in selectivity. Results reveal that adult males hold exclusive territories considerably smaller than those found in previous work conducted in “open” or large reserves. Similarly, results for the size of male versus female territories were also not in keeping with those from previous field studies, with males, rather than females, having the larger territory requirement. Terrain selection for both genders and age classes (adult and sub-adult) showed a strong preference for open grassland and avoidance of hill slope and riparian terrains. This research reveals white rhinoceros terrain selection preferences and how they influence range requirements in small, closed reserves. We conclude that this knowledge will be valuable in future white rhinoceros conservation management in small private game reserves, particularly in decisions surrounding removal of surplus individuals or augmentation of existing populations, calculation of reserve carrying capacity and future private reserve acquisition.

## 1.0 Introduction

Private game reserves in South Africa are becoming increasingly integral to the maintenance and enhancement of its biodiversity. These reserves are fenced (closed) and often small in comparison to those managed by the State Authority (South Africa National Parks). Among the largest state run reserves are the Kruger National Park and the Kgalagadi Transfrontier National Park which cover 19,485 km^2^ 9,591 km^2^ respectively [1], while 123km^2^ is stated by Sims-Castley et al., [2] as the average size for private game reserves in the Eastern Cape. As such small private games reserves require intensive land and livestock management, to both maximize their “game” carrying capacity for wildlife tourism purposes, and to minimise problems linked to over-grazing, poor nutrient status and imbalance between predator and prey numbers [3].

It has long been recognised that the majority of wild animals develop a gender-specific home range, usually in relation to age [4], and that the ranging behaviour and patterns that result are a consequence of individuals utilising locations in ways which benefit them [5]. We can therefore consider home range as non-random use of space that brings the animal back to the same areas repeatedly [5]. Consequently, access to these locations is driven by a number of factors, including the distribution of key resources (food, water, and shelter), travel costs within the overall range (influence of terrain and other environmental features) and seasonality and defence costs (access to mating opportunities). As a result, access to home ranges and the resources they provide, are a complex mix of social, gender and environmental cues [5].

The Southern white rhinoceros (*Ceratotherium simum*), promoted by the wildlife tourism industry as one of the “big five” animals to see whilst participating in wildlife tourism ventures, has become central to the appeal of many private game reserves in South Africa, and therefore their economic viability. As such, private game reserve managers benefit from detailed knowledge of white rhinoceros habitat selection preferences, which can be assessed from their ranging behaviours. This information has particular practical utility, in that it can provide insights into reserve carrying capacity, as an understanding of animal ranging behaviours enables managers to predict animal distribution patterns and consequently their impact on vegetation [6, 7, 8], on other species [9, 10, 11, 12, 13] andon their conspecifics [14, 15, 16, 17, 18].

In highly heterogeneous landscapes, basic habitat use/availability models may overestimate carrying capacities, especially when areas of preferred habitat are isolated by terrain that is energetically expensive to traverse [19]. This can be of particular importance to territory holding species such as the white rhinoceros, as the amount of total accessible attractive habitat may give a better indication of territory quality than total territory size or amount of high value habitat *per se* [8]. To date very little research has been conducted on terrain preference in white rhinoceros, although Perrin and Brereton-Stiles [20] did record selection against steeper gradients in a study conducted in Hluhluwe-Umfolozi Game Reserve.

Range size in the southern white rhinoceros has previously been demonstrated to vary, largely in relation to gender, resource availability and population density. Pienaar et al [21] concluded that in the south-west region of Kruger National Park range size of territorial males was between 6.2–13.8 km^2^, whilst for females the figure was 7.23–45.23 km^2^. Conway and Goodman [22] reported similar findings from Ndumu game reserve in South Africa, where range size of territorial males was between 2.5–13.9 km^2^, whilst for females the figure was 4.7–22.9 km^2^. For both these reserves rhinoceros densities per km^2^ were similar (0.5–1.4 and 0.6–1.8 respectively). However in Hluhluwe-Umfolozi, Owen-Smith [23] indicated that a higher population density (3–5.7 individuals per km^2^) resulted in a much reduced range size for males (0.75–2.6 km^2^) but an increase in female range size (8.9–20.5 km^2^). White et al., [5] who studied spatial patterns of white rhinoceros distribution in relation to breeding strategies in Hluhluwe-Umfolozi, indicated that female home range size was approximately 20 km^2^, with a core area of approximately 5 km^2^, whilst male territory equated to roughly that of female core area.

Currently then, much of our understanding of wild white rhinoceros ranging behaviour originates from either observations in unfenced savannah situations [4, 24] or from large fenced reserves [5, 19, 20, 25, 26]. Therefore a key element of this research was to gain insights to the carrying capacity of a highly successful (in terms of white rhinoceros productivity and survival), yet relatively small private game reserve, derived from adult and sub-adult male and female home range analysis and terrain preference. This is particularly important for the future conservation of the species, as the majority of private game reserves in South Africa and beyond, both currently and especially into the future, will fall within this size category. Central to this analysis is an understanding of the terrain choices made by individuals in establishing their territories and the implications this has for individual territory overlap. As a consequence of this analysis we were able to compare and contrast our findings obtained in a small reserve against those from “open” or much larger reserves.

## 2.0 Methods

### 2.1 Study site

The field site was located on a private game reserve in South Africa, Welgevonden Game Reserve (hereafter “the reserve”), with permission received from the land owners and conservation management staff to conduct this research. The reserve is situated in the Waterberg plateau in the Limpopo Province of South Africa (24°10’ - 24°25’S and 27°45’ - 27°56E) and classified as Waterberg Mountain Bushveld vegetation type [27]. The reserve forms part of the Waterberg Biosphere Reserve, which in its entirety covers in excess of 400km^2^. Two perennial rivers flow through the reserve, whilst a further two streams have their headwaters there. There are a large number (17) of man-made dams present on the reserve, an artefact of the farming activities that took place prior to the reserve’s formation. As a consequence there is a plentiful water supply across the reserve all year round, meaning all animals on the reserve do not need to make excursions outside of their established territories in order to access water. Of particular relevance to the research is the presence of a steep sided valley system which dissects the reserve, effectively splitting it into two in terms of the ability of white rhinoceros to traverse the landscape. As a result of this natural barrier to movement the rhinoceros were considered as two populations (north and south) in all analyses undertaken.

The terrain in the reserve is mainly mountainous, interspersed with a number of plateaus and open plains. The underlying geology of the reserve is ancient sandstone which consists of a succession of coarse, clastic sedimentary rock [28]. Due to the acidic nature of the soil, the predominant vegetation is tall grass with low nutritional value that can only support small numbers of herbivores [29] and is referred to as “sour” bushveld. The reserve, as classified by Acocks [30], falls under two veld types—Veld type 20, comprising the majority of the reserve, and Veld type 18, which has a much more limited distribution. Veld type 20 has a typical tree community largely comprised of *Burkea africana*, *Faurea saligna*, *Protea caffra*, *Englerophytum magalismontanum*, *Dombeya rotundifolia*, *Lannea discolour*, *Combretum molle*, *Combretum zeheri*, *Gardenia volkensii*, *Diplorhynchus condylocarpum*, *Ficus thonningii*, *Kirkia wilmsii*, *Ochna pulcra*, *Strychnos pungens*, *Elephantorrhiza burkei*, *Nuxia congesta*, *Dovyalsi zeyheri*, *Pseudolachnostylis maprouneifolia*, *Euclea crispa* and Grewia spp. In Veld type 18 the following tree species are commonly found: *Terminalia sericea*, *Pterocarpus rotundifolia*, *Peltophorum africanum*, *Ziziphus mucronata*, *Ozoroa paniculosa*, *Mundelea sericea* and *Syzygium cordatum*. The common grass species encountered on the reserve are *Schizachyrium sanguineum*, *Schizachyrium jeffreysii*, *Elionurus muticus*, *Loudetia simplex*, *Diheteropogon amplectans*, *Hyperthelia dissolute*, *Trachypogon spicatus*, *Panicum natlense*, *Bracharia nigropedata*, *eragrostis curvula*, *Eragrostis superba*, *Themeda triandra*, *Sporobolus pectinatus*, *Heteropogon contortus*, *Pogonarthia squarrosa*, *Melinis repens*, *Urelytrum agropyroides* and Aristadia spp. and are all indicative of “sour” bushveld i.e. they are of low grazing value [31].

**Fig 1:**
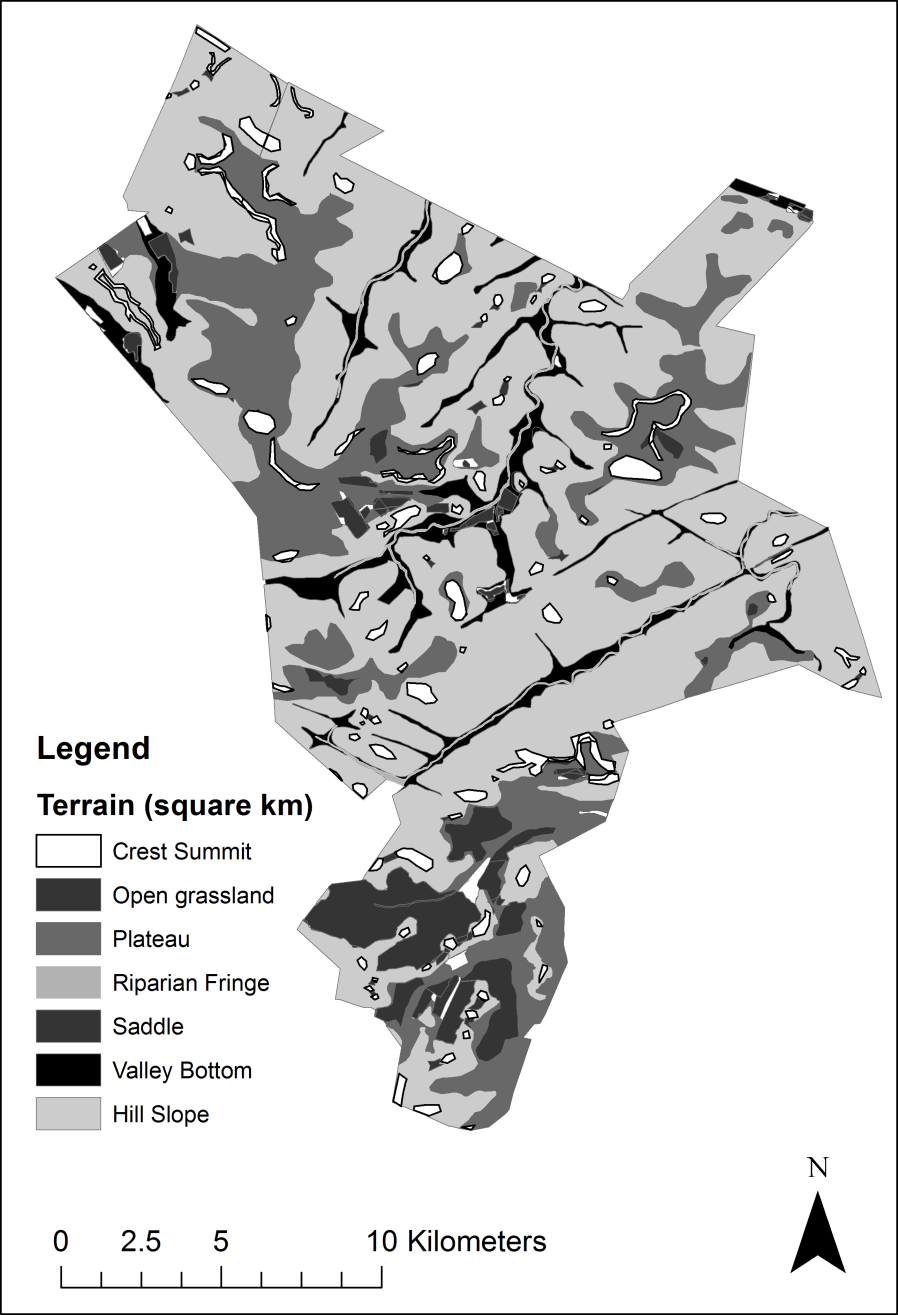
Terrain composition and terrain patch size (km^2^) in Welgevonden Game reserve adapted from Slater & Long [34].

Some areas of the reserve have dense thickets of shrubs and trees, whilst others which were previously used for citrus farming, have more nutrient rich soil and host a limited number of other slightly more nutritious grassland species. Throughout the reserve there are large amounts of rock protruding through the soil surface, further compounding the land’s ability to support large numbers of herbivores. For the purposes of this research the terrain of the reserve has been classified into seven different types; riparian, plateau, valley bottom, hill slope, crest/summit, old farmlands and plains (Fig 1). This classification was based upon a combination of the habitat types described in a white rhinoceros study in Kruger National Park by Pienaar [32], as well as comprehensive vegetation and land-use surveys conducted by the research team, existing land use maps and consultation with the reserve management. Together they were managed within the ArcGIS suite of software, employing ArcMap 10.3 [33] for all terrain and associated vegetation mapping.

### 2.2 Data collection

#### 2.2.1 Individual white rhinoceros identification

The majority of game reserves containing white rhinoceros in South Africa use an identikit classification system to identify individuals during survey and monitoring exercises. The one employed in this research was an adaptation of the Hitchens [35] identikit, originally constructed for black rhinoceros (*Diceros bicornis*) using horn morphology, hair orientation on tail, ear notching, scarring or other unique physical attributes (for example tail orientation) to identify individuals. Incorporated into this identikit is an age classification technique [36] which categorises individuals into one of six age classes (A to F), the age classes being A = 0–3 months, B = 3 months -1 year, C = 1–2 years, D = 2–3.5 years, E = 3.5–7 years and F = 7 years+. It should be noted that white rhinoceros age classification on the reserve is considered precise, a consequence of the long term monitoring programme and regular field surveys which have taken place historically.

For the purposes of this research we collected adult and sub-adult white rhinoceros distribution data over a 15 month period. Adults were defined as males and females which were classified by the field research team as “F” class, whilst sub-adults are classified as “E” class. For the purposes of analysis, adult and sub-adult males were differentiated between as it is the F males who demonstrate dominance over the E males, thus securing all mating opportunities. E and F females were not differentiated between in the analysis, as theoretically females reach reproductive age from 5–6 onwards and therefore referred to as adult females collectively from hereon in.

In this research we used three different data collection methods. First, all large mammal distributions on the reserve were intensively recorded from game vehicles by the research team during July/August 2011 and 2012 along six 10km line transects that coincide with the reserve road network. The transect lines incorporated all of the seven different terrain types (some transects covered only one terrain type while others covered multiple terrain types). Second, the research team conducted a year round monthly game transect across the entire reserve, recording all herbivore species as part of the annual monitoring and recording programme. Third, all *ad libitum* sightings of all age classes were also recorded by the research team whenever white rhinoceros were encountered throughout the duration of the study. These *ad libitum* sightings provided a significant proportion of all records obtained. For each of the three data collection methods, each time a white rhinoceros was located, the same protocol was observed. The individual was first identified from the field identikits. The GPS location of the vehicle was then obtained using either a hand held Trimble, (model Juno SA®) or from a bespoke “app” built by the authors for in-field real-time recording purposes (see WildKnowledge®). Finally, the distance to the animal and the bearing of the individual in relation to the vehicle were recorded using a range finder (Bushnell Sport 600®) and a compass.

#### 2.2.2 Range size

Individual range size was calculated using kernel density estimation analysis in Arcmap 10® [33] using the Kernel Density Estimator function. In order to determine areas of high use, the 50% probability density was calculated and plotted in the GIS to represent the more intensively used areas, or defended territories (50% kernel) of the individuals. The 95% probability densities are presented as the home ranges (95% kernel) of the individuals, which represent areas that while occupied are not used with the same intensity as the 50% kernel. From the kernel analysis area (ha) values were calculated for the territory and home range of each individual. To determine whether territory and range sizes varied between age groups and sexes, T-tests (two-tailed assuming unequal variances) were performed.

#### 2.2.3 Terrain selectivity

**Fig 2:**
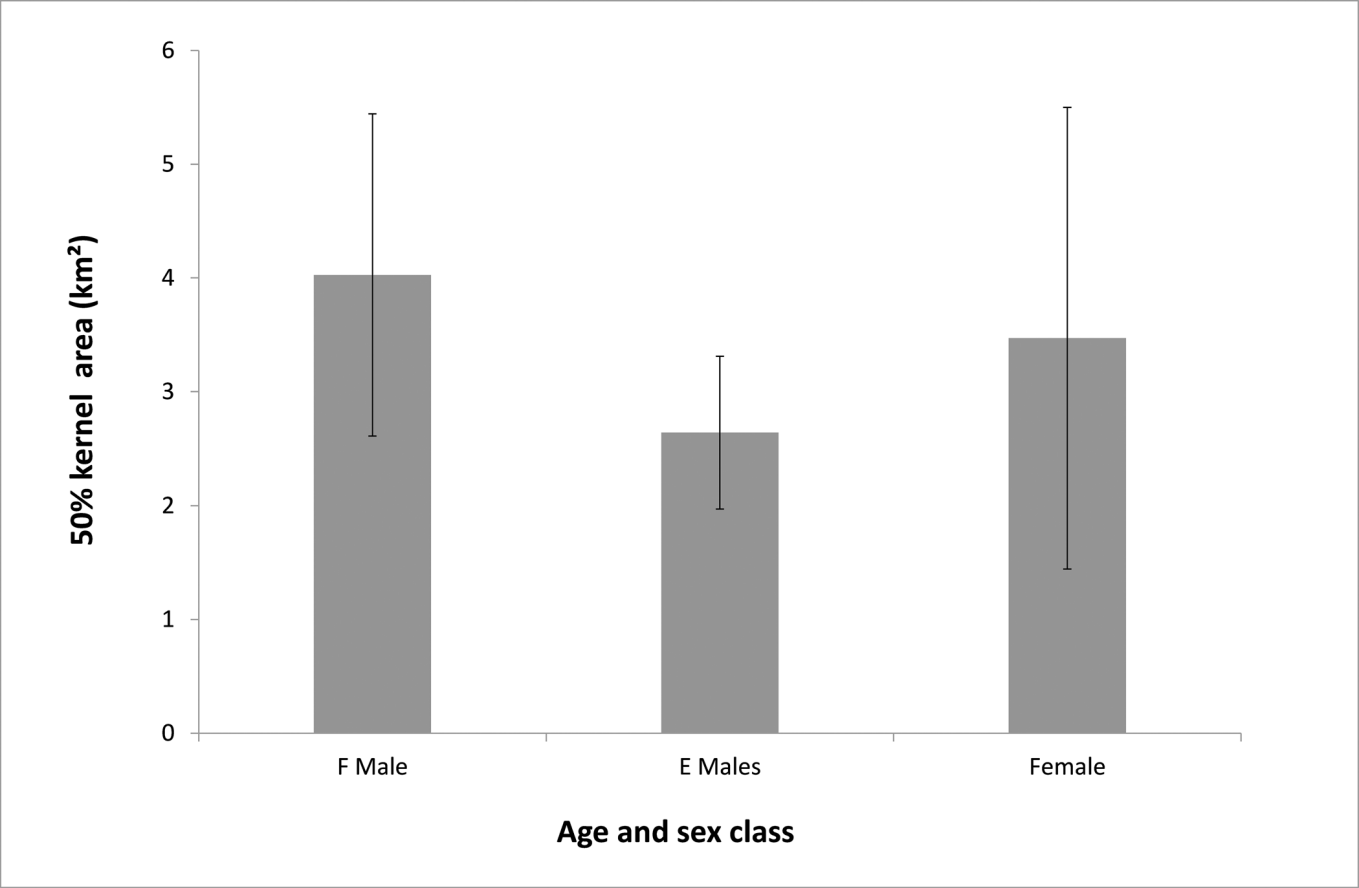
50% kernel size (km^2^) for adult and sub-adult males and females with 95% confidence intervals.

To perform the terrain selectivity analysis the calculated 50% and 95% kernels were used to extract terrain composition values from the reserve terrain map (Fig 2) for each individual. Initially rhinoceros density values were tested (ANOVA) against terrain classifications to determine whether there was a significant difference in the density distributions between different terrains.

Jacob’s correction [37] of Ivlev’s selectivity index [38] was then applied to the terrain composition of each of the E and F class male individuals and adult females in order to identify trends in selectivity. The Ivlev index measures the utilisation of a resource (in this case terrain) in relation to its availability [39]. However, terrain selectivity depends not only on the extent of selection, but also upon the relative abundance of the resource in relation to all other terrain types and can result in bias if not accounted for. This is accommodated by Jacob’s correction of the Ivlev index where:

Ivlev’s selectivity index

$$E=\frac{r-p}{r+p}$$

Where *r* is the proportion of terrain utilised and *p* is the proportion of that terrain available

Jacob’s correction:

$$J=\frac{r-p}{(r+p)-2rp}$$

Where *r* is the proportion of terrain utilised and *p* is the proportion of that terrain available.

Terrain type(s) that are selected in a larger proportion than would be expected from all those available are considered preferred (selectivity index values between 0.5 and +1), those which are under-represented are considered as being avoided (selectivity index values between -0.5 and -1) whilst terrains with selectivity index values between -0.5 and 0.5 were categorised as neither preferred nor avoided and consequently labelled ‘no preference’.

Terrain texture preferences were investigated by means of digital elevation data acquired from USGS (These data are distributed by the Land Processes Distributed Active Archive Center (LP DAAC), located at USGS/EROS, Sioux Falls, SD. http://lpdaac.usgs.gov) [40] and converted into a ruggosity metric: Relative topographic Index (RTP). Rhinoceros density values were tested against RTP to identify whether there was a relationship between the density distribution and RTP (Pearson’s correlation).

#### 2.2.4 Range ovelap analysis

The amount of core and home range overlap between adults (F male versus F male; F male versus E male; F male versus adult female, E male versus adult female and female versus female) was investigated. This allowed for the calculation of the amount of “exclusive” territory each individual presided over and to identify which individual males were preferentially selected by the females.

## 3.0 Results

### 3.1 Sightings

In total 632 sightings were recorded, comprising 147 for the five F class males (mean = 29.4, S.D. = 11.7), 118 for the five E class males (mean = 23.6, S.D. = 8.5) and 367 for the fifteen E and F class females (mean = 24.5, S.D = 15.4).

### 3.2 Range analysis

**Fig 3:**
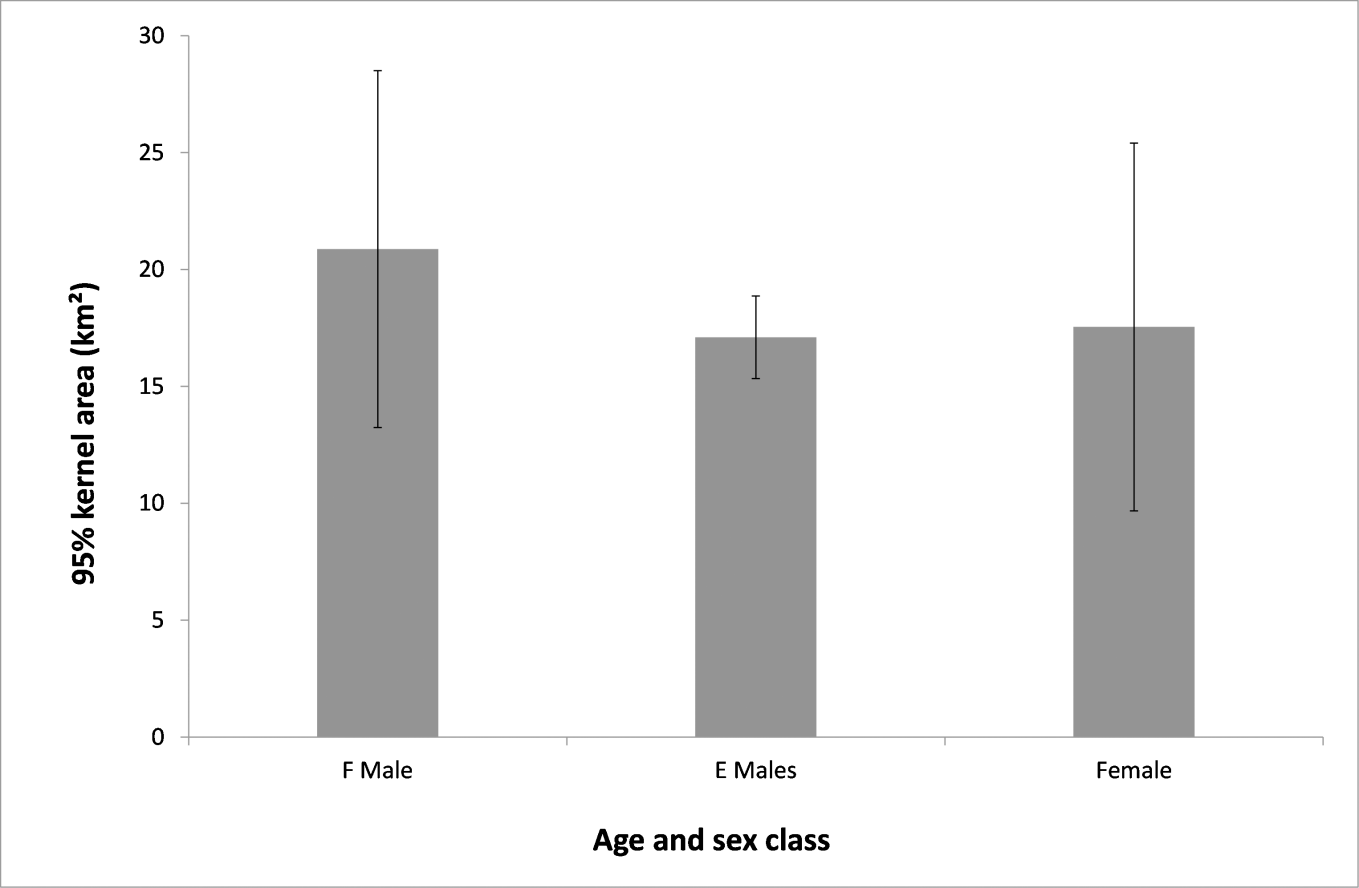
95% kernel size (km^2^) for adult and sub-adult males and females with 95% confidence intervals.

Range size analysis revealed that adult males (F class) had larger average 50% kernel size than both sub-adult males (E class) and females (Fig 2). Similarly, the 95% kernel size found for F males was larger than for both E males and females (Fig 3). Female 95% kernel sizes sit between F males and E males.

T-tests were performed to determine whether the difference in range size was significant between age groups (males) and sexes. All tests yielded non -significant results.

### 3.3 Terrain selectivity

Terrains were used differentially by adult males, sub-adult males and females with all ANOVA’s conducted on density distributions in the available terrains yielding significant results (p-values = 0.001) (S1 and S2 and S3 and S4 Tables). Subsequent analysis was conducted on individuals to determine which particular terrains were driving these differences.

Terrain selectivity for each adult was therefore calculated for individuals located in both the north and south of the reserve using the Ivlev selectivity index with Jacob’s correction (J) applied. The reserve was considered as two sub-units due to the presence of a steep sided valley which the rhinoceros would not readily negotiate.

In the northern part of the reserve open grassland was used selectively by six out of eight females, one F male and one E male (Table 1). Several individuals also displayed selectivity towards saddle (four females, one F male and one E male) and plateau (two females, one F male and two E males (Table 1). Hill slope was predominately avoided by E males and F males and half the adult female population in the Northern section of the reserve (Table 1). There was no consistent patternsin term of selectivity for Plateau in the F Males where one individual (Dolf) displayed strong selectivity, another no selectivity (Elvis) and the third male (Sydney) in the Northern section displayed strong avoidance of that terrain (Table 1).

**Table 1:** White rhinocerus (*Ceratotherium simum)* population in Northern sub-section of Welgevonden Game Reserve, South Africa—Ivlev’s selectivity index with Jacob’s correction (J) for terrain composition.

Sex / Age group	Indv.	Crest	Hill Slope	Open Grassland	Plateau	Riparian	Saddle	Valley
F Males	Dolf	-0.18	-0.76*	0.37	0.74**	0.49	0.02	-0.12
	Elvis	0.24	-0.28	0.03	0.27	0.26	0.49	-0.03
	Sydney	-1.00*	-0.96*	0.92**	-0.77**	0.98**	0.58**	0.08
E Males	Henrie	-0.02	-0.36	0.76**	-0.10	0.48	0.45	0.45
	M06	0.13	-0.56*	0.41	0.15	0.45	0.51**	0.41
	Riley	-0.33	-0.54*	0.33	0.50	-1.00*	0.47	0.24
	Ron	-0.41	-0.51*	0.37	0.53**	-1.00*	0.39	0.13
	Shaun	-0.40	-0.62*	0.37	0.60**	-1.00*	0.48	0.18
Females	Anika	-0.13	-0.55*	0.60**	0.49	-1.00*	0.43	0.07
	Mama- sita	0.06	-0.44	0.73**	-0.02	0.43	0.46	0.45
	Mandy	0.06	-0.66*	0.60**	0.57**	-0.12	0.87**	-0.33
	Rosy	0.11	-0.44	0.75**	-0.06	0.38	0.52**	0.43
	Sally	0.22	-0.61*	0.82**	0.10	0.35	0.63**	0.37
	Sophie	-0.08	-0.32	0.52**	0.10	0.42	0.70**	0.30
	Stompie	0.30	-0.36	0.39	0.37	-0.89*	0.38	-0.33
	Susan	-0.40	-0.56*	0.40	0.58**	-1.00*	0.41	0.05

J < -0.5 = Avoidance*; -0.5 < J > 0.5 = No selectivity; J > 0.5 = Selectivity**

In the southern section of the reserve, patterns of selectivity were similar to those obtained for the northern section. The majority of individuals demonstrated selectivity towards open grasslands and saddle (Table 2). Interestingly two individuals (F male and a female) were found to occupy riparian terrain selectively. In the southern section of the reserve, hill slope was the terrain that was most consistently avoided among individuals (Table 2). The two F males in this sourthern section of the reserve demonstrated different levels of selectivity towards the riparian terrain, with one (Sharky) strongly avoiding it and the other (Shrek) selecting it (Table 2).

**Table 2:** White rhinocerus (*Ceratotherium simum)* population in Southern sub-section of Welgevonden Game Reserve, South Africa—Ivlev’s selectivity index with Jacob’s correction (J) for terrain composition.

Sex / Age group	Indv.	Crest	Hill Slope	Open Grassland	Plateau	Riparian	Saddle	Valley Bottom
F Males	Sharky	-0.02	-0.679*	0.505**	0.299	-1.00*	0.562**	-1.00
	Shrek	0.085	-0.558*	0.561**	0.016	0.569**	0.503**	-1.00
E Males	Otto	0.084	-0.479	-0.162	-0.607*	-0.988	-0.976*	-1.00
Females	Betty	-0.126	-0.614*	0.56**	0.145	0.182	0.562**	-1.00
	Cloe	0.009	-0.552*	0.672**	-0.268	0.336	0.456	-1.00
	Fiona	-0.008	-0.698*	0.500	0.325	-1.00*	0.555**	-1.00
	Gill	-0.054	-0.491	0.677**	-0.412	0.037	0.502**	-1.00
	Lucy	-0.037	-0.532*	0.559**	0.043	0.153	0.279	-1.00
	Nandi	0.008	-0.697*	0.662**	0.035	-1.00*	0.478	-1.00
	Rubee	0.071	-0.555*	0.524**	0.07	0.598**	0.596**	-1.00

J < -0.5 = Avoidance*; -0.5 < J > 0.5 = No selectivity; J > 0.5 = Selectivity**

### 3.4 Range exclusivity

#### 3.4.1 F males

All adult males maintained completely exclusive territories (50% kernel) from other adult males (Table 3). All adult males but one maintained 100% exclusivity over their territory from sub-adult males.

**Table 3:** Exclusive core territory (50% kernel) and home range (95% kernel) for F class class white rhinocerus males (*Ceratotherium simum)* males (km^2^ & %) in Welgevonden Game Reserve.

	Exclusive Territory (50%)				Home range (95%)			
	From other F males		From E males		From other F males		From E males	
Individual	Km^2^	%	Km^2^	%	Km^2^	%	Km^2^	%
**Dolf**	5.17	100	5.17	100	20.55	95	9.51	44
**Elvis**	3.78	100	3.78	100	18.45	100	18.45	100
**Sharkie**	2.88	100	2.89	100	7.45	78	6.57	69
**Shrek**	2.51	100	2.51	100	22.49	91	10.51	43
**Sydney**	5.78	100	0	48	28.98	96	13.06	43
**Mean**	3.46	100	3.43	90	19.58	92	11.62	60
**S.D**	2.11	0	1.08	23.1	7.85	8.5	4.47	25.0

The amount of home range exclusivity within the adult male population was high (Mean = 92%) while the percentage of adult males’ home range from sub-adult males varied. Some did not overlap at all with sub-adults (Elvis) while most had some degree of overlap (mean = 60%) (Table 3).

#### 3.4.2 E males

The amount of E male core territory and home range, in relation to both other E males and F males showed a high degree of variability. Some individuals (M06 and Otto) maintained total exclusion from all other E males in terms of exclusive territory, with one (Otto) also maintaining this level of exclusion for his overall home range (Table 4).

**Table 4:** Exclusive core territory (50% kernel) and home range (95% kernel) for E class white rhinocerus (*Ceratotherium simum)* males (km^2^ & %) in Welgevonden Game Reserve.

	Exclusive Territory (50%)				Home range (95%)			
	From F males		From other E males		From F males		From other E males	
Individual	Km^2^	%	Km^2^	%	Km^2^	%	Km^2^	%
**Henrie**	0.08	2	1.00	30	3.05	17	9.10	51
**M06**	1.29	56	2.31	100	0	0	5.56	38
**Otto**	1.29	66	1.94	100	2.59	14	18.47	100
**Riley**	0.67	29	0.83	35	0	0	1.97	12
**Ron**	1.47	40	3.06	84	0	0	5.60	29
**Shaun**	0.95	41	0.40	17	0	0	2.29	14
**Mean**	0.96	39	1.59	61	0.94	5	7.17	41
**ST dev**	0.51	22.3	1.01	37.6	1.46	8.0	6.12	32.5

The remainder maintained varying amounts of exclusive territory (17–84%) and home range (12–51%). E males also displayed considerable variability in the amounts of exclusive territory (2–66%) and home range (0–17%) they were able to maintain within the reserve.

#### 3.4.3 Females

Females were found to hold no exclusive territory from adult males (F males), while percentages varied greatly with sub-adult (E) males (Table 5). Over half maintained no exclusive territories from sub-adult males while others had between 52–100% overlap with young males. One individual (Stompie) had completely exclusive territory from all E-males (Table 5).

**Table 5:** Female white rhinocerus (*Ceratotherium simum)* exclusive core territory (50% kernel) and home range (95% kernel) from F and E males in Welgevenden Game Reserve.

	Core Territory (50%)				Home range (95%)			
	From F males		From E males		From F males		From E males	
Individual	Area (Km^2^)	%	Area (Km^2^)	%	Area (Km^2^)	%	Area (Km^2^)	%
**Anika**	0	0	0	0	1.92	7	19.13	74
**Betty**	0	0	1.59	92	0	0	5.32	37
**Chloe**	0	0	0	0	0.20	2	1.84	14
**Fiona**	0	0	1.07	81	0	0	6.99	62
**Lucy**	0	0	2.58	59	4.64	21	11.10	51
**Mamasita**	0	0	0	0	1.37	10	1.08	8
**Mandy**	0	0	0.60	10	8.47	50	12.42	73
**Nandi**	0	0	2.75	52	0	0	4.19	58
**Rosy**	0	0	0	0	0.44	3	0	0
**Rubee**	0	0	0	0	2.29	11	5.57	28
**Sally**	0	0	0	0	0	0	0	0
**Sophie**	0	0	0	0	13.13	36	17.05	47
**Stompie**	0	0	5.83	100	13.07	46	20.80	73
**Susan**	0	0	0	0	0.10	1	0.65	4
**Gill**	0	0	0	0	0.72	9	1.66	20
**Mean**	0	0	0.96	26	3.09	13	7.19	37
**S.D**	0	0	1.66	39	4.67	17	7.20	28

## 4.0 Discussion

The primary objective of this research was to explore the relationship, both between and within, gender of adult and sub-adult white rhinoceros in a small contained (fenced) game reserve, in terms of range analysis and terrain preference. This we considered important as small private game reserves are increasingly becoming the stronghold of white rhinoceros distribution, as they are estimated to hold up to 25% of rhinoceros in South Africa [41], given the considerable poaching pressures experienced in the larger, open game reserves. Recent data [42] on poaching of rhinoceros supports this assertion, indicating that 1,215 rhinoceros are known to have been poached in 2014 and 1,175 in 2015 in South Africa alone, with the majority of these incidents taking place in the larger reserves [4]. In response, wildlife managers are taking steps to translocate individuals from poaching hotspots such as Kruger National Park to smaller private game reserves in to order fo better ensure their protection [43].

Our results revealed that the F-class males held exclusive territories between 1.14 km^2^ and 5.17km^2^, with a mean of 3.46 km^2^ (S.D. = 2.11 km^2^), whilst for E-class males the exclusive territory size lay between 0.4km^2^ and 3.06km^2^ with a mean of 1.45km^2^ (S.D. = 1.06 km^2^). Given the more pronounced territorial nature of F-class males as compared to E-class [4], these differences in exclusive territory sizes were expected, although we were unable to directly compare our findings with that of previous authors as none have differentiated between the two age classes. These exclusive territory values were however considerably lower than the findings from many of the “open” reserves [22, 26] but greater than for Hluhluwe-Umfolozi [20].

Analysis of the adult female territory data revealed that (with one exception) individuals were not maintaining any exclusive territory from males and therefore their territories overlapped completely with males of both age classes. Females did however maintain a degree of exclusivity from males within their home ranges, with many individuals showing a reasonably high retention of this aspect of their overall territory requirement, particularly in relation to E-males (mean = 37%, SD = 28%). This result indicates that the females have a component of their overall territory requirements within which they are not overlapping with the E-males. This is a consequence of E-class males sharing significant proportions of their 50% kernels with each other, rather than establishing their own territories in the ‘spare’ capacity on the reserve.

Our results for the size of male versus female territories were not in keeping with those of previous authors [5, 25, 44] working in open or large reserves, who all demonstrate that female home ranges are significantly larger than males. Whilst the differences in male and female range size from our closed reserve were not statistically significant, they are of interest. In open reserves, females will have to travel long distances in order to interact with multiple males, the interaction serving to identify and subsequently mate with the most suitable breeding partners. In our closed situation, the females were constrained in their ability to traverse the landscape as a consequence of the presence of tracts of unsuitable terrain reducing opportunities for mate selectivity. Ultimately there will be genetic costs to this constraint and conservation management implications for augmentation programmes in particular (a common practice in private game reserves), with careful thought needed to be given to where new incumbents are placed into the landscape and “who” therefore they are likely able to interact with.

In small game reserves, reserve managers should be aware of the degree of overlap territory owning (F-class) males will tolerate from sub-adult (E-class) males in both their core and home ranges, as this acts as a surrogate for the likelihood of injuries sustained from territorial battles. These battles prove costly to reserve managers as they must provide veterinary care for the injured animals, with the injuries potentially impacting upon future off-take of surplus animals. As a consequence we considered the territorial relationship between both E and F-class males. Rather unsurprisingly, the F-class males did not tolerate any E-class males within their core (50% kernel) territory but did allow E-class males into their overall home (95% kernel) range (Mean = 40%, SD = f 25%). Interestingly, although the reserve has been very successful in terms of youngsters born, the rhinoceros density in the reserve is 0.15 rhino per km^2^ (based on 2012 census of 58 rhinoceros) and is considerably lower than in other reserves e.g. Hluhluwe-Umfolozi which had > 3 rhinoceros per km^2^ [20), whilst both Kruger National Park and Ndumu both supported >0.5 rhinoceros/km^2^ [21, 22]. Here we note that the density is kept low by the reserve management team (who translocate surplus animals) but it does raise the question as to why such low densities are not resulting in larger individual territories, particularly for females? We propose that this is a function of the heterogeneous nature of the terrain mosaic of the reserve and that this would be a careful consideration when planning to establish new populations in similar sized vegetation and terrain comprised reserves. In addition to this, the plentiful all year round supply of water across the reserve negates the requirement for an individual to travel long distances to find water, thus decreasing the size of the home territory[45].

Due to the presence of a steep sided valley that transects the reserve which the rhinoceros would be very unlikely to negotiate, we analysed terrain selection in two distinct sub-units (north and south). In both the north and south, males and females showed a strong preference for open grassland and avoidance of hill slope and riparian terrains. Hill slopes would be very energetically expensive to traverse, so even if there was spare attractive territory beyond the hill slopes, females would be unlikely to occupy them, and without females, they would offer very little value to a male territory holder. Similarly, riparian terrains in the reserve tend to be step-sided, presenting the rhino with similar access conditions as encountered on hill slopes. Both hill slope and riparian terrain types tend to be the more heavily wooded and our results compare favourably with that of White *et al* [5], who demonstrate that dense and medium woodland proved unattractive in terms of habitat composition, making up less than 10% of overall territory for both males and females. That said, direct comparison between our results and those from previous authors proved difficult, as the majority of previous work has utilised habitat rather than terrain descriptors. This was particularly evident when comparing our findings for terrain preference against the findings of White *et al* [5] who indicate that open woodland accounted for over 60% of habitat composition in male and female rhinoceros territories, whilst this research indicates open grassland and saddle terrains to be particularly favoured.

Closer examination of the Ivlev values revealed that some individuals in the north (Sydney = 0.924, Henrie = 0.757, Rosy = 0.749, Mamasita = 0.822) were demonstrating a very strong preference for open grassland habitat. However, in the south the Ivlev values for open grassland were not as pronounced (Shrek = 0.561, Gill = 0.677, Nandi = 0.662). This was a consequence of differences in preferred and “no preference” terrain availability. The north of the reserve is a much more heterogeneous mix of the various terrain types, resulting in preferred terrain types often being enclosed by avoided terrain types, particularly hill slope. This was not the case in the south, where preferred terrain was often contiguous with “no preference” terrain, notably plateau and saddle.

Both males and females demonstrated complete avoidance of valley bottom in the south. This a consequence of the very small and highly fragmented units of this terrain type and the sheer nature of the immediate terrain adjacent to the valley bottom making it difficult for the rhinoceros to traverse. In the north, where valley bottom terrain was relatively abundant, the terrain type was still not considered preferential by both genders, with selectivity values indicating no preference in every instance.

We conclude that in small game reserves understanding both range requirements and the influence of terrain has considerable utility in the conservation management of white rhinoceros. Much of our knowledge to date has been accumulated from studies undertaken at a time when white rhinoceros densities and population growth rates were considerably higher than they are today. We conducted our study in a reserve whose size reflects a typical private enterprise, whilst there are undoubtedly behavioural, particularly mate selection, influences upon white rhinoceros distribution patterns, this research has provided revealing analysis of white rhinoceros terrain selection preferences and how they influence range requirements in small, closed reserves. This we suggest will be valuable in the future conservation management of the species in decisions surrounding removal of surplus individuals or augmentation of the current reserve population, calculation of reserve carrying capacity and future reserve design and subsequent management.

## Supporting Information

S1 Table ANOVA outputs for density distributions in the available terrains yielding significant results (p-value = 0.001) of F male, E Male and Female (E & F merged) rhinoceros in Welgevonden Game Reserve. (DOCX)

S2 Table Post hoc tukey tests for F class male rhinoceros density values were tested against terrain classifications to determine whether there was a significant difference in the density distributions between different terrains in Welgevonden Game Reserve. (DOCX)

S3 Table Post hoc tukey tests for E class male rhinoceros density values were tested against terrain classifications to determine whether there was a significant difference in the density distributions between different terrains in Welgevonden Game Reserve. (DOCX)

S4 Table Post hoc tukey tests for female (F and E class) rhinoceros density values were tested against terrain classifications to determine whether there was a significant difference in the density distributions between different terrains in Welgevonden Game Reserve. (DOCX)
