# Supplementary material for: Range Analysis and Terrain Preference of Adult Southern White Rhinoceros (Ceratotherium simum) in a South African Private Game Reserve: Insights into Carrying Capacity and Future Management
Source: PLoS One. 2016 Sep 13;11(9):e0161724. doi: 10.1371/journal.pone.0161724 (PMC5021330; doi:10.1371/journal.pone.0161724)
Supplement: S1 Table — (DOCX) [file pone.0161724.s001.docx]

Appendix 1: ANOVA outputs for density distributions in the available terrains yielding significant results (p-value = 0.001) of F male, E Male and Female (E & F merged) rhinoceros in Welgevonden Game Reserve

| Demographic group | F | p-value |
| --- | --- | --- |
| F Males | 24.72 | 9.01E-07 |
| E Males | 10.69 | 1.35E-12 |
| Females | 37.85 | <2e-16 |
