# Supplementary material for: Range Analysis and Terrain Preference of Adult Southern White Rhinoceros (Ceratotherium simum) in a South African Private Game Reserve: Insights into Carrying Capacity and Future Management
Source: PLoS One. 2016 Sep 13;11(9):e0161724. doi: 10.1371/journal.pone.0161724 (PMC5021330; doi:10.1371/journal.pone.0161724)
Supplement: S2 Table — (DOCX) [file pone.0161724.s002.docx]

Appendix 2: Post hoc tukey tests for F class male rhinoceros density values were tested against terrain classifications to determine whether there was a significant difference in the density distributions between different terrains in Welgevonden Game Reserve

| Pairwise comparison | difference | lower | upper | p-values | Significance |
| --- | --- | --- | --- | --- | --- |
| 2 to 1 | -1.43721 | -7.50834 | 4.633925 | 0.99638 | N.S |
| 3 to 1 | -0.45749 | -6.39601 | 5.48104 | 0.999998 | N.S |
| 4 to 1 | -1.98996 | -8.24156 | 4.261629 | 0.978569 | N.S |
| 5 to 1 | 1.080217 | -4.90853 | 7.068965 | 0.999369 | N.S |
| 6 to 1 | 1.484422 | -5.51243 | 8.481269 | 0.998192 | N.S |
| 7 to 1 | -2.5313 | -8.48741 | 3.424817 | 0.901148 | N.S |
| 8 to 1 | -0.63351 | -7.3387 | 6.071668 | 0.999992 | N.S |
| 3 to 2 | 0.979724 | -0.49939 | 2.458843 | 0.472043 | N.S |
| 4 to 2 | -0.55276 | -3.00309 | 1.897577 | 0.997328 | N.S |
| 5 to 2 | 2.517426 | 0.84805 | 4.186802 | 0.000151 | 5 less than 2 |
| 6 to 2 | 2.921631 | -1.06304 | 6.906299 | 0.334558 | N.S |
| 7 to 2 | -1.09409 | -2.6423 | 0.454131 | 0.38379 | N.S |
| 8 to 2 | 0.803695 | -2.64321 | 4.250604 | 0.996709 | N.S |
| 4 to 3 | -1.53248 | -3.63285 | 0.567888 | 0.341032 | N.S |
| 5 to 3 | 1.537702 | 0.444893 | 2.630511 | 0.00058 | 5 less than 3 |
| 6 to 3 | 1.941907 | -1.83764 | 5.721457 | 0.771747 | N.S |
| 7 to 3 | -2.07381 | -2.97069 | -1.17693 | 0 | 3 less than 7 |
| 8 to 3 | -0.17603 | -3.38361 | 3.031555 | 1 | N.S |
| 5 to 4 | 3.070181 | 0.831751 | 5.30861 | 0.000913 | 4 less than 5 |
| 6 to 4 | 3.474386 | -0.78018 | 7.728952 | 0.20398 | N.S |
| 7 to 4 | -0.54133 | -2.69092 | 1.608257 | 0.994684 | N.S |
| 8 to 4 | 1.35645 | -2.3992 | 5.112103 | 0.956861 | N.S |
| 6 to 5 | 0.404205 | -3.45378 | 4.262187 | 0.999984 | N.S |
| 7 to 5 | -3.61151 | -4.79617 | -2.42685 | 0 | 7 less than 5 |
| 8 to 5 | -1.71373 | -5.01337 | 1.585909 | 0.761801 | N.S |
| 7 to 6 | -4.01572 | -7.82284 | -0.20859 | 0.030368 | 7 less than 6 |
| 8 to 6 | -2.11794 | -7.01471 | 2.778837 | 0.892508 | N.S |
| 8 to 7 | 1.897781 | -1.34225 | 5.137809 | 0.632173 | N.S |

1 = saddle; 2 = other; 3 = plains; 4 = Hill slope; 5 = valley bottom; 6= Riparian fringe; 7= Plateau; 8= Crest summit; N.S = not significant; A less than B = terrain A is used than terrain B
