# Supplementary material for: Range Analysis and Terrain Preference of Adult Southern White Rhinoceros (Ceratotherium simum) in a South African Private Game Reserve: Insights into Carrying Capacity and Future Management
Source: PLoS One. 2016 Sep 13;11(9):e0161724. doi: 10.1371/journal.pone.0161724 (PMC5021330; doi:10.1371/journal.pone.0161724)
Supplement: S3 Table — (DOCX) [file pone.0161724.s003.docx]

Appendix 3: Post hoc tukey tests for E class male rhinoceros density values were tested against terrain classifications to determine whether there was a significant difference in the density distributions between different terrains in Welgevonden Game Reserve

| Pairwise comparison | difference | lower | upper | p-values | Significance |
| --- | --- | --- | --- | --- | --- |
| 2 to 1 | 1.142303 | -1.88805 | 4.172652 | 0.945915 | N.S |
| 3 to 1 | 1.012282 | -1.94426 | 3.968822 | 0.967751 | N.S |
| 4 to 1 | -0.36852 | -3.38949 | 2.652456 | 0.999954 | N.S |
| 5 to 1 | 0.908681 | -2.06744 | 3.884805 | 0.983142 | N.S |
| 6 to 1 | 1.254923 | -2.22668 | 4.73653 | 0.957343 | N.S |
| 7 to 1 | 0.257716 | -2.70528 | 3.220717 | 0.999996 | N.S |
| 8 to 1 | -1.33799 | -4.59104 | 1.915057 | 0.91574 | N.S |
| 3 to 2 | -0.13002 | -0.90951 | 0.649465 | 0.999624 | N.S |
| 4 to 2 | -1.51082 | -2.50719 | -0.51445 | 0.000134 | N.S |
| 5 to 2 | -0.23362 | -1.08438 | 0.617134 | 0.990994 | N.S |
| 6 to 2 | 0.112621 | -1.88439 | 2.109632 | 1 | N.S |
| 7 to 2 | -0.88459 | -1.68823 | -0.08095 | 0.019479 | 7 less than 2 |
| 8 to 2 | -2.4803 | -4.04516 | -0.91543 | 5.06E-05 | 8 less than 2 |
| 4 to 3 | -1.3808 | -2.12301 | -0.6386 | 7E-07 | 4 less than 3 |
| 5 to 3 | -0.1036 | -0.63454 | 0.427337 | 0.998946 | N.S |
| 6 to 3 | 0.242641 | -1.64049 | 2.125768 | 0.999933 | N.S |
| 7 to 3 | -0.75457 | -1.20617 | -0.30296 | 1.42E-05 | 7 less than 3 |
| 8 to 3 | -2.35028 | -3.76693 | -0.93362 | 1.69E-05 | 8 less than 3 |
| 5 to 4 | 1.2772 | 0.460465 | 2.093935 | 6.86E-05 | 4 less than 3 |
| 6 to 4 | 1.623443 | -0.35931 | 3.6062 | 0.201161 | N.S |
| 7 to 4 | 0.626236 | -0.1413 | 1.39377 | 0.20496 | N.S |
| 8 to 4 | -0.96947 | -2.51611 | 0.57716 | 0.546214 | N.S |
| 6 to 5 | 0.346243 | -1.56748 | 2.25997 | 0.999357 | N.S |
| 7 to 5 | -0.65096 | -1.21677 | -0.08516 | 0.011731 | 7 more than 5 |
| 8 to 5 | -2.24667 | -3.70376 | -0.78959 | 9.36E-05 | 8 less than 5 |
| 7 to 6 | -0.99721 | -2.89046 | 0.896046 | 0.748473 | N.S |
| 8 to 6 | -2.59292 | -4.91399 | -0.27185 | 0.016531 | 8 less than 6 |
| 8 to 7 | -1.59571 | -3.0258 | -0.16562 | 0.016751 | 8 less than 7 |

1 = saddle; 2 = other; 3 = plains; 4 = Hill slope; 5 = valley bottom; 6= Riparian fringe; 7= Plateau; 8= Crest summit; N.S = not significant; A less than B = terrain A is used than terrain B
