# Supplementary material for: Range Analysis and Terrain Preference of Adult Southern White Rhinoceros (Ceratotherium simum) in a South African Private Game Reserve: Insights into Carrying Capacity and Future Management
Source: PLoS One. 2016 Sep 13;11(9):e0161724. doi: 10.1371/journal.pone.0161724 (PMC5021330; doi:10.1371/journal.pone.0161724)
Supplement: S4 Table — (DOCX) [file pone.0161724.s004.docx]

Appendix 3: Post hoc tukey tests for female (F and E class) rhinoceros density values were tested against terrain classifications to determine whether there was a significant difference in the density distributions between different terrains in Welgevonden Game Reserve

| Pairwise comparison | Difference | Lower | Upper | p-values | Significance |
| --- | --- | --- | --- | --- | --- |
| 2 to 1 | -0.96947 | -2.51611 | 0.57716 | 0.546214 | N.S |
| 3 to 1 | 0.119665 | -1.90646 | 2.145786 | 1 | N.S |
| 4 to 1 | -1.50124 | -3.55646 | 0.553987 | 0.34026 | N.S |
| 5 to 1 | 0.36245 | -1.6778 | 2.402698 | 0.999434 | N.S |
| 6 to 1 | 0.470118 | -1.91784 | 2.858073 | 0.998892 | N.S |
| 7 to 1 | -0.71071 | -2.73961 | 1.318189 | 0.963661 | N.S |
| 8 to 1 | -1.72329 | -3.87182 | 0.425234 | 0.224342 | N.S |
| 3 to 2 | 0.008155 | -0.48828 | 0.504587 | 1 | N.S |
| 4 to 2 | -1.61275 | -2.21709 | -1.00841 | 0 | 4 less than 2 |
| 5 to 2 | 0.250939 | -0.30032 | 0.802203 | 0.864359 | N.S |
| 6 to 2 | 0.358608 | -0.99919 | 1.716404 | 0.992935 | N.S |
| 7 to 2 | -0.82222 | -1.32986 | -0.31457 | 2.94E-05 | 7 less than 2 |
| 8 to 2 | -1.8348 | -2.70512 | -0.96449 | 0 | 8 less than 2 |
| 4 to 3 | -1.6209 | -2.0486 | -1.1932 | 0 | 4 less than 3 |
| 5 to 3 | 0.242785 | -0.10591 | 0.591483 | 0.404759 | N.S |
| 6 to 3 | 0.350453 | -0.93846 | 1.639371 | 0.991573 | N.S |
| 7 to 3 | -0.83037 | -1.10497 | -0.55578 | 0 | 7 less than 3 |
| 8 to 3 | -1.84296 | -2.60135 | -1.08457 | 0 | 8 less than 3 |
| 5 to 4 | 1.863687 | 1.373408 | 2.353967 | 0 | 4 less than5 |
| 6 to 4 | 1.971355 | 0.637154 | 3.305557 | 0.000223 | 4 less than 6 |
| 7 to 4 | 0.790529 | 0.349864 | 1.231195 | 1.9E-06 | 4 less than 7 |
| 8 to 4 | -0.22206 | -1.05508 | 0.610971 | 0.992526 | N.S |
| 6 to 5 | 0.107668 | -1.20335 | 1.418681 | 0.999997 | N.S |
| 7 to 5 | -1.07316 | -1.43764 | -0.70867 | 0 | 7 less than 5 |
| 8 to 5 | -2.08574 | -2.8811 | -1.29038 | 0 | 8 less than 6 |
| 7 to 6 | -1.18083 | -2.4741 | 0.112452 | 0.102747 | N.S |
| 8 to 6 | -2.19341 | -3.66729 | -0.71953 | 0.000193 | 8 less than 6 |
| 8 to 7 | -1.01259 | -1.77836 | -0.24681 | 0.00166 | 8 less than 7 |

1 = saddle; 2 = other; 3 = plains; 4 = Hill slope; 5 = valley bottom; 6= Riparian fringe; 7= Plateau; 8= Crest summit; N.S = not significant; A less than B = terrain A is used than terrain B
